# Letter from AIChE President

**DOI:** 10.1002/btm2.10006

**Published:** 2016-05-27

**Authors:** Gregory Stephanopoulos

**Affiliations:** ^1^ 2016 AIChE President, Past Chair, SBE, W.H. Dow Professor of Chemical Engineering and Biotechnology, Massachusetts Institute of Technology

Quantitative approaches and application of fundamentals are the currency of chemical engineers. Our accomplishments in pharmaceuticals, food, materials, and energy are unsurpassed in providing solutions critical to societal well‐being. For the last 40 years, spurred by the biological revolution and the expansion of the pharmaceutical industry into biologics, chemical engineering—as in any robust discipline—began to produce the top engineers with expanded, interdisciplinary proficiencies in biology‐based skills. Witness, as a result, the explosive growth of the biopharmaceutical, biotech, and biofuels industries, driven by chemical and biological engineering approaches.

More than a decade ago, the American Institute of Chemical Engineers (AIChE) founded the Society for Biological Engineering (SBE) to further capture and expand the “new biology” as a foundational science, along with chemistry and chemical engineering. Not surprisingly, as with previous chemical engineering accomplishments, the quantitative and integrative methods employed by these “new biology” engineers have brought a deeper, fundamental understanding of biomolecular, cellular, and tissue events and processes that have significant clinical merit. Engineers in this space have honed a number of approaches—metabolic engineering, bioinformatics, computational modeling, genetic engineering, synthetic biology—to aid and accelerate the identification and formation of therapies for human health. This explosion of ideas is converted into actual therapeutic modalities that are being validated by National Institutes of Health (NIH) programs funded and targeted specifically for these translational science activities. For example, the NIH's National Center for Advancing Translational Sciences, founded in 2011, speaks directly to “transforming knowledge into treatments.”

The allied fields encompassed within translational medicine involve chemical, biochemical, biological, biomolecular, and biomedical engineers as critical players in its intellectual infrastructure. So, SBE and AIChE are excited to advance and recognize developments in this space with yet another initiative, this new journal, *Bioengineering & Translational Medicine. Bioengineering & Translational Medicine* adds to the arsenal of opportunities that SBE and AIChE are building for members across the disciplines.

We welcome all who are contributing to the birth of this new publication. I am delighted and gratified that this strategic initiative not only expands the mission and exceptional opportunities SBE promises, but that it also has come to fruition during my tenure as AIChE president.

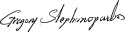


Gregory Stephanopoulos
2016 AIChE President
Past Chair, SBE
W.H. Dow Professor of Chemical Engineering and Biotechnology
*Massachusetts Institute of Technology*

